# A system for coordinated analysis of translational readthrough and nonsense-mediated mRNA decay

**DOI:** 10.1371/journal.pone.0173980

**Published:** 2017-03-21

**Authors:** Stacey L. Baker, J. Robert Hogg

**Affiliations:** Biochemistry and Biophysics Center, National Heart, Lung, and Blood Institute, National Institutes of Health, Bethesda, Maryland, United States of America; Korea University, REPUBLIC OF KOREA

## Abstract

The nonsense-mediated mRNA decay (NMD) pathway degrades mRNAs containing premature termination codons, limiting the expression of potentially deleterious truncated proteins. This activity positions the pathway as a regulator of the severity of genetic diseases caused by nonsense mutations. Because many genetic diseases result from nonsense alleles, therapeutics inducing readthrough of premature termination codons and/or inhibition of NMD have been of great interest. Several means of enhancing translational readthrough have been reported to concomitantly inhibit NMD efficiency, but tools for systematic analysis of mammalian NMD inhibition by translational readthrough are lacking. Here, we introduce a system that allows concurrent analysis of translational readthrough and mRNA decay. We use this system to show that diverse readthrough-promoting RNA elements have similar capacities to inhibit NMD. Further, we provide evidence that the level of translational readthrough required for protection from NMD depends on the distance of the suppressed termination codon from the end of the mRNA.

## Introduction

The nonsense-mediated mRNA decay pathway is responsible for recognizing and degrading aberrant mRNAs undergoing premature translation termination while also serving as a major regulator of normal cellular gene expression [1]. To achieve specificity, the NMD pathway is thought to determine whether a translation termination event is distant from the end of the transcript, a condition associated with, but not unique to, nonsense mutations. According to a unified model for NMD, a major determinant of transcript susceptibility to decay is 3’UTR length [2]. In vertebrates, the pathway further uses the presence of an exon-junction complex (EJC) downstream of the termination codon (TC) as a strong signal that the transcript contains a premature TC and should be degraded [3].

To maintain accuracy and efficiency of mRNA decay, NMD proteins engage in a complex and dynamic network of protein-protein and protein-RNA interactions [4]. The decision to decay is carried out by a conserved set of core NMD proteins, which work with additional factors to identify potential decay substrates, recruit decay enzymes, and ultimately degrade the mRNA. The process of NMD is coordinated by the highly conserved UPF1 superfamily I RNA helicase. UPF1 makes extensive backbone contacts to RNA, enabling high-affinity but sequence-nonspecific RNA binding [5]. Elongating ribosomes appear to efficiently clear UPF1 from coding sequences, resulting in preferential accumulation of UPF1 on long 3’UTRs [6–8]. In addition to its intrinsic RNA binding activity, UPF1 engages with mRNPs via protein-protein interactions. Of particular importance is direct recognition of translation termination by UPF1, through interactions with translation release factors eRF1 and eRF3 [9]. UPF1 can be joined in this complex, termed SURF (SMG1-UPF1-eRF1-eRF3) by the SMG1 kinase, which phosphorylates UPF1, leading to the recruitment and/or activity of the SMG6 endonuclease, the SMG5/7 heterodimer, and additional decay proteins including decapping factors [10–16]. In addition, UPF1’s ATPase, phosphorylation, and decay-promoting activity is stimulated by UPF2, which interacts with UPF3b to link UPF1 to the EJC [5, 17].

Due to their potentially drastic effects on protein sequence, structure, and function, nonsense alleles are a particularly deleterious class of mutations, as illustrated by estimates that they make up approximately 11% of mutations associated with human genetic disease [18, 19]. It has been widely hypothesized that the NMD pathway may have evolved to ameliorate the phenotypic consequences of truncated proteins; however, degradation of mutant transcripts may not always be advantageous [20]. For example, reductions in levels of truncated protein by NMD may exacerbate the effects of certain Duchenne muscular dystrophy and Tay-Sachs disease alleles [21–23]. Therefore, both activators and inhibitors of NMD may be therapeutically beneficial, depending on the nature of the genetic lesion.

Extensive evidence indicates that translational readthrough induced by a variety of mechanisms (including cis-acting RNA structures, suppressor tRNAs, selenocysteine incorporation, and small molecule termination inhibitors) can inhibit NMD [6, 24–33]. Further, diverse viruses may exploit readthrough to protect their RNAs from NMD [34]. In many of these studies, NMD inhibition was observed with even relatively low levels of readthrough (i.e. 1–5%), such as that which can be attained by pharmacological inhibition of translation termination. However, it remains unknown whether readthrough caused by distinct mechanisms will inhibit NMD to the same extent. Further, the inhibition of NMD by readthrough presents opportunities for mechanistic dissection of the NMD pathway and development of improved therapeutics.

Our previous findings suggest that translational readthrough can inhibit nonsense-mediated mRNA decay at multiple steps, depending on features of the substrate mRNA and the rate of readthrough. Frequent readthrough can displace UPF1 from the mRNA downstream of the suppressed TC, while inefficient readthrough allows UPF1 association but blocks the initiation of decay at a subsequent rate-limiting step [6]. In order to rigorously study the effects of translational readthrough on mRNA decay, we established a set of reporter mRNAs allowing simultaneous analysis of readthrough efficiency and mRNP protein composition. Here, we use this system to show a correlation between readthrough efficiency and NMD inhibition by three distinct RNA elements, the Moloney murine leukemia virus pseudoknot (MLVPK) [35], a hairpin structure from the Colorado Tick Fever Virus (CTFVHP) [36], and a nonsense-mutation associated with junctional epidermolysis bullosa previously observed to undergo inefficient termination [37]. By varying the length of the open reading frame downstream of the suppressed TC, we present evidence that the ability of readthrough to protect potential NMD targets depends on the length of the conditionally translated downstream ORF. Together, these results establish a flexible platform for investigation of the impact of translational recoding on NMD.

## Materials and methods

### Plasmids

The sequence inserted into the HindIII and ApaI sites of pcDNA 3.1 (pc2iFPgr) or pcTET2βwtβ (pcTET2iFPgr; [38]) is shown in S1 Fig. The indicated recoding elements were cloned using oligonucleotides (IDTDNA) into the SpeI and BamHI sites of the 2FP cassette. MLVPK sequences (13PK6, [39]) and the CTFV-106 element, containing 16 nt upstream and 87 nt downstream of the VP9 UGA stop codon from CTFV segment 9 mRNA, were as described [36]. For 3’UTR extension experiments, the NanoLuc luciferase coding sequence was amplified by PCR from the pNL1.1 vector (Promega) and inserted into the BamHI site of the indicated pcTET2iFPgr vector variant by homologous recombination, removing the mCherry TC. The pcTET2iFPgr variant containing a codon-optimized HIV RNase H was previously described [32].

### Immunoblotting

For immunoblotting of whole cell lysates and purified mRNPs, rabbit anti-UPF1 (H-300, Santa Cruz) and mouse anti-PABC1 (10E10, Abcam) were used. Secondary antibodies labeled with IRDye 680 or IrDye 800 (Rockland) were detected using an Odyssey imaging system (Li-Cor).

### Readthrough assays

293 Tet-off cells (Clontech) plated in triplicate at a density of 1x10^5^ cells per well in 24-well PureCoat Amine plates (BD) were transfected with 250 ng pcTET2iFP plasmid, using 0.5 μL Turbofect (ThermoFisher) per well. Two days after transfection, cells were washed in 1xPBS and lysed in 1X Passive Lysis Buffer (Promega). Lysates were transferred to 96-well plates and fluorescence was monitored with a Tecan Infinite F200 plate reader. Readthrough efficiency was calculated by normalizing the mCherry:GFP ratio from pcTET2iFP constructs containing the indicated stop codon contexts with the mCherry:GFP ratio derived from control constructs lacking stop codons between the GFP and mCherry ORFs. Readthrough values reported are the average of values obtained from three or more independent experiments performed on different days.

### mRNA decay assays

mRNA decay assays were performed as described, with minor modifications[40, 41]. HeLa Tet-off cells (Clontech) were plated at a density of 5x10^5^ cells per 60 mm plate and transfected with a mixture of 800 ng pcTET2iFP, 200 ng pcDNA GFP TP [6], and 500 ng empty pcDNA 3.1, using 3 μL Turbofect. Transcription from pcTET2iFP vectors was repressed with 2 ng/mL doxycycline (Sigma-Aldrich). The next day, cells were trypsinized, resuspended in 4 mL total volume, and distributed equally to four wells of a 12-well plate. After 24 additional hours, cells were washed with 1xPBS, and media was replaced with DMEM containing 10% tetracycline-free FBS (Clontech). Four hours later, 1 μg/mL doxycycline was added to halt transcription, cells were incubated for an additional 30 minutes, and total RNA was harvested using Trizol (ThermoFisher) at the indicated intervals. For RNA decay assays involving UPF1 knockdown, HeLa Tet-off cells were first reverse-transfected with non-targeting (AN2, Ambion/ThermoFisher) or UPF1-specific siRNAs [42] in 24-well plates as described [41], allowed to incubate for 24 hours, and subsequently transfected with Turbofect as above. Decay assays were conducted approximately 72 hours after the initial siRNA transfection.

### mRNP purifications

For mRNP purification, whole cell extracts of transiently transfected 293 cells were incubated with the ZZ-tev-PP7CP (tandem protein A-tagged PP7 coat protein) and rabbit IgG-conjugated M-270 Dynabeads (Dynal/ThermoFisher) as described [6]. Bound RNA and protein were eluted from beads in LDS sample buffer (ThermoFisher).

## Results

### A unified reporter system for analysis of translational read-through and mRNA decay

**Fig 1:**
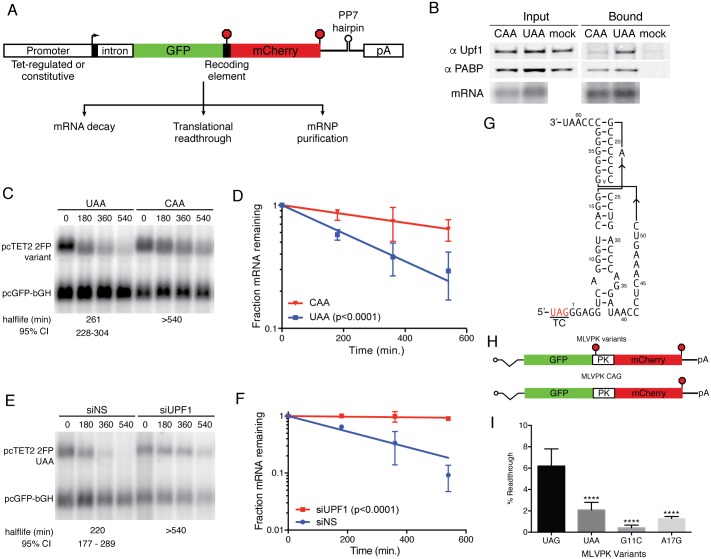
Unified system for assays of recoding, mRNA decay, and mRNP composition. (a) Schematic of constructs used to express PP7-tagged mRNAs encoding dual-fluorescent protein reporters. Stop signs indicate possible positions of termination codons; pA refers to bovine growth hormone polyadenylation signal. (b) Immunoblot of UPF1 and PABPC1 present in input extracts (left) or PP7-based affinity purifications containing mRNAs with (UAA) or without (CAA) a stop codon between the GFP and mCherry ORFs. Bottom, mRNA recovery was monitored by northern blot. Mock purifications lacking tagged RNAs were performed in parallel to assess background recovery of proteins. (c) Constructs containing an efficient UAA TC or no TC (CAA) between the GFP and mCherry ORFs were used for pulse-chase mRNA decay assays. Northern blotting with a probe against GFP sequence was used to quantify abundance of tetracycline-regulated experimental mRNAs (pcTET2 2FP) and constitutively expressed control mRNAs (pcGFP-bGH). RNA half-lives corresponding to best-fit lines to semi-log plots of RNA abundance data and 95% confidence intervals (CI) are listed (n = 3). (d) Semi-log plot of mRNA decay assays shown in C. Error bars indicate +/- SD (n = 3); p-value derived from ANCOVA analysis comparing CAA and UAA. (e) Decay assay of mRNAs containing a UAA TC in cells treated with non-silencing control siRNAs or anti-UPF1 siRNAs. RNA half-lives corresponding to best-fit lines to semi-log plots of RNA abundance data and 95% confidence intervals (CI) are listed (n = 3). (f) Semi-log plot of mRNA decay assays shown in E. Error bars indicate +/- SD (n = 3); p-value derived from ANCOVA analysis comparing siNT and siUPF1. (g) Schematic of MLVPK RNA secondary structure, indicating the position of the wild-type UAG TC [39]. (h) Schematic of mRNAs (top) containing a single upstream TC regulated by a MLVPK variant, (middle) containing the MLVPK sequence but lacking a TC, and (bottom) containing the MLVPK sequence and an additional in-frame UAA TC. Positions of the pseudoknot sequences are indicated by PK. (i) Relative readthrough efficiencies calculated from GFP and mCherry levels produced by mRNAs containing the indicated MLVPK variants. Error bars indicate ± SD (n = 3); ****: p<0.0001 in one-way ANOVA analysis comparing the indicated samples to UAG. See Materials and methods for details of readthrough efficiency calculations.

We first established vectors for reporter mRNA expression containing a tetracycline-regulated minimal CMV promoter widely used for pulse-chase studies of mRNA decay [43], followed by an efficiently spliced intron and open reading frames (ORFs) encoding the fluorescent proteins GFP and mCherry (Fig 1A). The two fluorescent protein ORFs are encoded in the same reading frame, separated by a short spacer into which termination codons and translational recoding elements can be inserted as desired (see S1 Fig for sequence). Finally, these mRNAs can be engineered to contain a single copy of the RNA hairpin binding site of the Pseudomonas phage 7 coat protein (PP7cp), previously established as a powerful tool for isolation of endogenously assembled ribonucleoprotein complexes [6, 44].

### Affinity purification of mRNPs

Previously, we showed that UPF1 non-specifically binds mRNAs, leading to its preferential accumulation on long 3’UTRs [6]. Using the PP7cp binding site incorporated into the reporter mRNAs, we affinity-purified mRNPs containing the indicated tagged mRNAs. Following mRNP isolation, we immunoblotted co-purifying proteins to detect endogenous UPF1 and cytoplasmic poly-A binding protein 1 (PABPC1). Consistent with studies of other well-established NMD reporters, mRNAs containing stop codons upstream of the mCherry ORF recruited higher levels of UPF1 than those with distal stop codons (Fig 1B) [6, 45].

### Suitability of reporters for analysis of mRNA decay

A primary aim of this work is to measure rates of readthrough and NMD with a unified reporter system. To verify that the mCherry sequence conferred decay susceptibility when used as a 3'UTR, we performed pulse-chase mRNA decay assays using reporters in which a UAA termination codon was inserted downstream of the GFP ORF, preventing translation of the mCherry ORF (Fig 1C). As expected, the mRNAs in which translation was terminated upstream of the mCherry sequence were unstable, with half-lives of approximately 260 minutes (Fig 1C and 1D). In contrast, mRNAs in which no TC was included between the GFP and mCherry ORFs were highly stable (CAA; Fig 1C and 1D). Depletion of the essential NMD factor UPF1 with specific siRNAs rescued stability of the UAA-containing reporter, demonstrating that the mCherry-derived 3'UTR renders transcripts are susceptible to NMD (Fig 1E and 1F).

### Accurate and sensitive detection of translational readthrough with dual-fluorescent reporters

As in widely used luciferase-based readthrough and frameshifting reporters, translational recoding events can be assessed via the ratio of production of the protein encoded by the downstream ORF (here, mCherry) relative to expression from the upstream ORF (GFP) [46, 47]. Readthrough can then be quantified by normalizing the fluorescent protein expression to the signal derived from matched control constructs containing single nucleotide changes that convert the termination codon to a sense codon (see Materials and methods for details). To determine whether the dual-fluorescent protein constructs faithfully measure rates of translational readthrough, we inserted the MLVPK sequence between the GFP and mCherry ORFs (Fig 1G). Experimental constructs contained the wild-type UAG termination codon, the UAA termination codon, or a series of point mutations in the pseudoknot structure previously determined to impair readthrough to varying extents (Fig 1H, top)[39]. Throughout, controls in which the TC was mutated to CAG were used to determine the signal derived from maximal expression of the readthrough product (Fig 1H, bottom). The indicated vectors were transiently transfected into 293 Tet-off cells in the absence of doxycycline, and GFP and mCherry fluorescence was monitored in cell lysates after 48 hours. The extent of readthrough measured with the 2FP constructs consistently recapitulated previous measurements reported in the literature (Fig 1I, see also below). Moreover, this method proved to be sensitive, as we used it to reproducibly quantify the less than 1% readthrough frequency promoted by the MLVPK G11C mutant. Together, these findings indicate that the dual-fluorescent protein constructs introduced here are suitable for simultaneous biochemical and functional analysis of translational readthrough and mRNA decay.

### Relationship between 3’UTR length and readthrough-mediated stabilization

**Fig 2:**
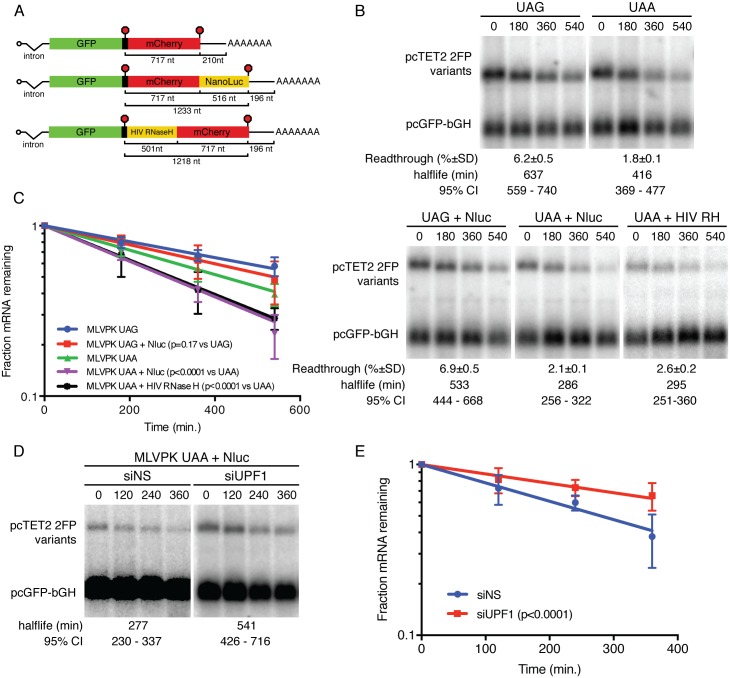
Relationship between readthrough-mediated stabilization and downstream ORF length. (a) NanoLuc or HIV RNase H sequences were added to dual-fluorescent reporter mRNAs in the indicated positions. (b) Decay assays of mRNAs in which UGA or UAA TCs following the GFP ORF were modulated by the MLVPK and NanoLuc (Nluc) or HIV RNase H (HIV RH) sequences were added as indicated. RNA half-lives corresponding to best-fit lines to semi-log plots of RNA abundance data and 95% confidence intervals are listed (n = 3). Readthrough efficiencies (mean +/- SD; n = 3) for each construct are indicated below the blot panels. (c) Semi-log plot of mRNA decay assays shown in B. Error bars indicate +/- SD (n = 3); p-values derived from ANCOVA analysis. (d) Decay assays of mCherry-NanoLuc reporter mRNAs as in A. Cells were treated with control siRNAs (siNS) or UPF1 siRNAs (siUPF1) as indicated. Half-lives are represented as in B. (e) Quantification of mRNA decay assays shown in D; note reduced duration of timecourse vs. C (360 min.). Error bars indicate +/- SD (n = 3); p-values derived from ANCOVA analysis.

To further investigate the interplay among 3’UTR length, readthrough, and NMD, we modified the dual-fluorescent reporter mRNAs to contain an additional in-frame coding sequence downstream of the mCherry ORF. Specifically, we incorporated the NanoLuc luciferase protein, encoded by ~500 bp codon-optimized sequence, due to its rapid folding properties, small size, and potential use as a secondary measure of readthrough efficiency [48]. No termination codon was included between the mCherry and NanoLuc ORFs, meaning that any readthrough of the termination codon upstream of mCherry would result in a GFP-mCherry-NanoLuc fusion protein (Fig 2A). To test the ability of readthrough to rescue stability of these mRNAs, we inserted either the wild-type MLVPK element (~5% readthrough) or the UAA MLVPK variant (~2% readthrough) and performed fluorescent readthrough assays to verify that addition of the NanoLuc sequence did not affect readthrough efficiency (Fig 2B; readthrough efficiencies are listed below each panel). mRNA decay assays revealed that ~6% readthrough of the extended mCherry-NanoLuc sequence supported mRNA half-lives slightly, but not significantly, shorter than those observed for constructs containing only the mCherry sequence downstream of the wild-type MLVPK (Fig 2B and 2C). In contrast, mRNAs with the mCherry-NanoLuc 3’UTR undergoing ~2% readthrough were markedly less stable than their shorter counterparts (compare UAA to UAA + NanoLuc samples). RNAi-mediated UPF1 depletion rescued stability of UAA + NanoLuc reporter mRNAs, confirming that the observed decay was due to NMD (Fig 2D and 2E).

We have previously shown that binding of PTBP1 in the vicinity of TCs can protect mRNAs from long 3’UTR-mediated NMD [41]. To control for possible unintended sequence-based effects of the addition of NanoLuc above, we inserted a distinct 3’UTR extension into the mCherry-derived 3’UTR and conducted readthrough and mRNA decay assays. We recently used dual-fluorescent protein reporter mRNAs containing a codon-optimised HIV RNase H ORF upstream of the mCherry sequences as a control to analyze the readthrough-promoting activity of the M-MLV RNase H (Fig 2A, bottom) [32]. Whereas the MLV RNase H protein contains an insertion responsible for binding eRF1 and suppressing termination, the HIV RNase H protein lacks this feature and its associated activity. Suggesting that the requirement for increased readthrough efficiency on longer conditional ORFs observed here is not specific to the NanoLuc sequence used or its placement in the 3’UTR, mRNAs with the UAA MLVPK variant and NanoLuc extension (516 nt) have nearly identical half-lives to RNAs in which the HIV RNase H (501 nt) was used to extend the conditionally translated portion of the 3’UTR (Fig 2B and 2C).

### Analysis of the colorado tick fever virus readthrough-promoting element

**Fig 3:**
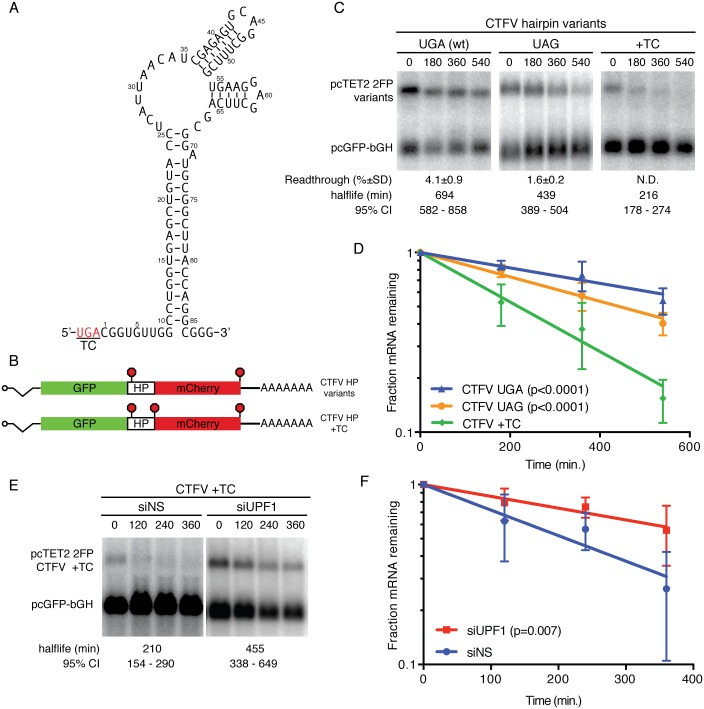
Distinct readthrough-promoting elements inhibit NMD. (a) Schematic of the proposed CTFV readthrough-promoting RNA hairpin structure (HP) [36]. (b) CTFV HP variants were inserted into dual-flourescent reporters. As a “no-readthrough” control, an additional TC was inserted downstream of the HP sequence (CTFV HP +TC). (c) Decay assays of mRNAs containing the indicated CTFV HP variants. RNA half-lives corresponding to best-fit lines to semi-log plots of RNA abundance data and 95% confidence intervals are listed (n = 3). Readthrough efficiencies (mean +/- SD; n = 3) for each construct are indicated below the blot panels. (d) Semi-log plot of mRNA decay assays shown in C. Error bars indicate +/- SD (n = 3); p-values derived from ANCOVA analysis. (e) Decay assays of CTFV +TC reporter mRNAs. Cells were treated with control siRNAs (siNS) or UPF1 siRNAs (siUPF1) as indicated. Half-lives are represented as in C. (f) Quantification of mRNA decay assays shown in E; note reduced duration of timecourse vs. D (360 min.). Error bars indicate +/- SD (n = 4); p-values derived from ANCOVA analysis.

In order to analyze the effects of distinct recoding RNA elements on readthrough-associated RNA stabilization, we first compared the activities of the well-characterized MLVPK to an RNA hairpin derived from the unrelated Colorado tick fever virus (CTFV; Fig 3A). We chose this element in part because a variety of mutants impairing its activity have been thoroughly characterized, allowing interrogation of its anti-NMD activities across a range of readthrough efficiencies [36]. Introduction of the CTFVHP to the 2FP reporter constructs reproducibly caused 4–5% readthrough in our assays (Fig 3B and 3C; readthrough efficiencies are listed below each panel in C). In the initial characterization of the CTFV element, replacement of the wild-type UGA TC with UAG abolished readthrough activity, but we observed substantial (~2%) readthrough of the UAG variant (Fig 3C) [36]. It is not clear what accounts for this discrepancy, but it is possible that local sequence context influences the ability of the hairpin to promote readthrough. Nevertheless, this set of constructs exhibits readthrough induced by the CTFV element at efficiencies from 2% to 5%, enabling direct comparisons with MLVPK-containing constructs supporting readthrough over this range.

In mRNA decay assays performed as described above, the wild-type CTFVHP fully stabilized the reporter transcripts (Fig 3C and 3D). Moreover, the CTFVHP variant in which the UAG codon was replaced with UGA showed intermediate levels of stability, consistent with the ~2% readthrough supported by this variant. As with the MLVPK-containing mRNAs (Fig 1F and 1G), addition of a UAA TC immediately downstream of the CTFVHP to abolish readthrough of the mCherry ORF led to rapid mRNA decay (Fig 3C and 3D; CTFV +TC). To confirm that the observed decay was due to NMD, we assayed the decay of the CTFV +TC variant in cells treated with control siRNAs (Fig 3E and 3F; siNS) or UPF1 siRNAs (siUPF1). As expected based on our previous finding that the mCherry-derived 3’UTR confers NMD susceptibility (Fig 1E and 1F), UPF1 depletion rescued stability of the CTFVHP variant unable to undergo readthrough. These findings show that protection of transcripts from NMD is not a unique property of the MLVPK sequence and that distinct readthrough-promoting RNAs can protect mRNAs from NMD.

### Readthrough of a premature termination codon associated with junctional epidermolysis bullosa

**Fig 4:**
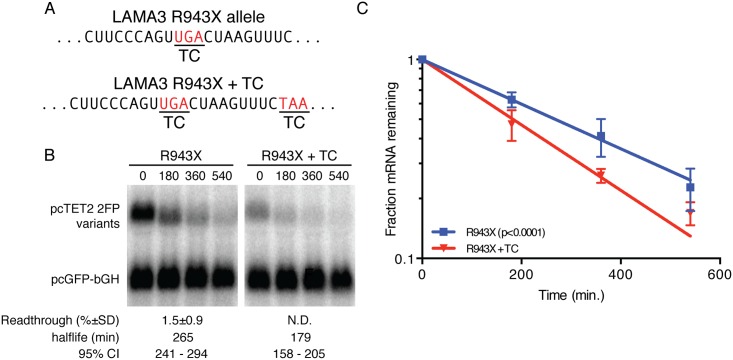
mRNA stabilization by readthrough of a pathogenic nonsense allele. (a) Sequence of the LAMA3 R943X allele [37]. (b) Decay assays of mRNAs containing the indicated LAMA3-derived sequence variants. Readthrough efficiencies (mean +/- SD; n = 3) for each construct are indicated below the blot panels. (c) Semi-log plot of mRNA decay assays shown in C. Error bars indicate +/- SD (n = 3); p-value derived from ANCOVA analysis.

The efficiency of translation termination is determined not only by stop codon identity but also by the mRNA sequences immediately flanking it [30, 49–54]. UGA tends to be least efficient of the three termination codons, and this effect can be augmented greatly by an unfavorable sequence context. Importantly, decreased termination efficiency due to sequences flanking a nonsense mutation has been observed to substantially increase production of full-length protein in patients carrying nonsense alleles [37, 55]. In one such case, a patient with junctional epidermolysis bullosa was found to have two distinct nonsense mutations in the laminin subunit **α**-3 gene (LAMA3) that were predicted to cause infant mortality in combination (R943X and R1159X; Fig 4A) [37]. However, readthrough of the AGT TGA CTA sequence of the R943X allele presumably inhibited NMD, increasing levels of mRNA and protein produced from this copy of LAMA3 and permitting development through at least five years of age. Consistent with a previous report, insertion of the R943X sequence into the dual-fluorescent reporter system described here resulted in approximately 1% readthrough (Fig 4B; readthrough efficiency is listed below R943X panel).

### Distinct readthrough elements support comparable inhibition of NMD

It is possible that the highly structured MLVPK and CTFV elements could physically occlude termination and/or NMD factors from the ribosome, providing an additional level of protection beyond that afforded by inefficient translation termination *per se*. To test this possibility, we examined the decay rates of mRNAs containing the unstructured LAMA3 R943X sequence described above, along with a control containing an additional in-frame TC. RNAs containing the inefficient LAMA3 R943X-derived TC were significantly stabilized relative to mRNAs unable to undergo readthrough (Fig 4B and 4C). Moreover, the degree of stabilization upon ~1% readthrough of this construct was consistent with expectations based on the observed relationship between readthrough and mRNA halflife for MLVPK and CTFV constructs. Together, these observations suggest that structure downstream of the suppressed TC is unnecessary for effective NMD inhibition.

## Discussion

Small molecule inhibitors of translation termination are particularly attractive candidates for treatment of diverse genetic diseases [18, 56]. Readthrough-promoting compounds may have beneficial effects in two major ways: increased production of full-length protein from each mRNA and inhibition of NMD to increase mRNA abundance. Because efficient NMD of nonsense-containing transcripts can substantially limit the pool of mRNA available for translation, interventions that promote concurrent translational readthrough and RNA stabilization are likely to be more effective than those only capable of the former. While pairing a readthrough-promoting drug with an anti-NMD drug has led to increased full-length protein production in cell culture and model systems, the broad impact of the NMD pathway on gene expression and known cytotoxicity of NMD inhibition may preclude such a therapeutic approach [57–59]. An alternative strategy might be to enhance stability of specific target RNAs by maximizing readthrough of nonsense mutations.

NMD efficiency has previously been observed to correlate with 3’UTR length in organisms from yeast to man [40, 60–65]. We have previously suggested a model in which 3’UTR length leads to enhanced accumulation of UPF1 on 3’UTRs, which in turn favors decay initiation [6]. Alternatively, it has been suggested that 3’UTR length impacts NMD because PABPC1 can less successfully compete for release factor binding when spatially separated from the terminating ribosome by an extended 3’UTR [60, 63, 66]. Translational readthrough affords a unique opportunity to probe this system, as it disrupts the process of decay even though effective 3’UTR length remains the same during most translation termination events. Therefore, readthrough would be expected to perturb the ability of UPF1 to assemble on mRNPs and engage in productive decay complexes without affecting the average distance of PABPC1 to the termination codon.

Translational readthrough can potentially affect the initiation of NMD in multiple ways. Alteration of the decoding process by structured RNAs or unfavorable sequence contexts may also affect the ability of UPF1 to interact with release factors at the terminating ribosome. Alternatively, translocation of the ribosome through the mRNP downstream of the termination codon could disrupt association of UPF1 with the 3’UTR and/or the formation of NMD complexes required for commitment to RNA decay. Previously, we have observed that ~5% readthrough of an artificial GAPDH-derived 3’UTR of approximately 560 nt efficiently displaces UPF1, resulting in inaccurate sensing of 3’UTR length [6]. However, less efficient readthrough was found to allow UPF1 binding to mRNPs but maintain robust NMD inhibition. This finding implied that the commitment to RNA decay is kinetically separable from the association of UPF1 with mRNPs, and, further, that readthrough may disrupt complex assembly, post-translational modifications, or other events required for decay.

An important step in understanding the utility of readthrough-promoting RNAs and small molecules for inhibition of NMD is determining how much readthrough is required to stabilize various transcripts. In human cells, two prominent NMD susceptibility factors have been identified: 1) the presence of one or more exon junctions more than 55 nt downstream of a termination codon and 2) 3’UTR length. Previously, we have shown that transcripts undergoing accelerated EJC-dependent NMD can be effectively stabilized by low levels of translational readthrough [6]. After a single readthrough event, the EJC(s) will be displaced from the RNA, leaving the RNA subject to NMD based on 3’UTR length alone. Here, we focus on the relationship between 3’UTR length and readthrough efficiency, showing that increasing 3’UTR length reduces the effectiveness of readthrough in inhibiting decay. Genome-wide mapping of SMG6 cleavage sites suggests that decay effector complexes assemble and act near the termination codon [67–70]. Since the rate of translation elongation through complexes assembled near the termination codon should not vary with 3’UTR length, extending 3’UTR length presumably affects either the kinetics or likelihood of formation of an mRNP committed to decay. Therefore, our data are consistent with the model that binding of UPF1 along the length of the 3’UTR is an important determinant of decay induction kinetics.

## Supporting information

S1 Fig Sequence of dual-fluorescent reporters. Top, schematic of the dual-fluorescent readthrough and NMD reporters. Bottom, sequence of a reporter gene containing an in-frame UAA termination codon (underlined) between the GFP (green text) and mCherry (red text) ORFs. The intron and PP7 hairpin sequences are indicated in purple and orange, respectively.
(TIF)
