# Supplementary figures and images for: A system for coordinated analysis of translational readthrough and nonsense-mediated mRNA decay
Source: PLoS One. 2017 Mar 21;12(3):e0173980. doi: 10.1371/journal.pone.0173980 (PMC5360307; doi:10.1371/journal.pone.0173980)

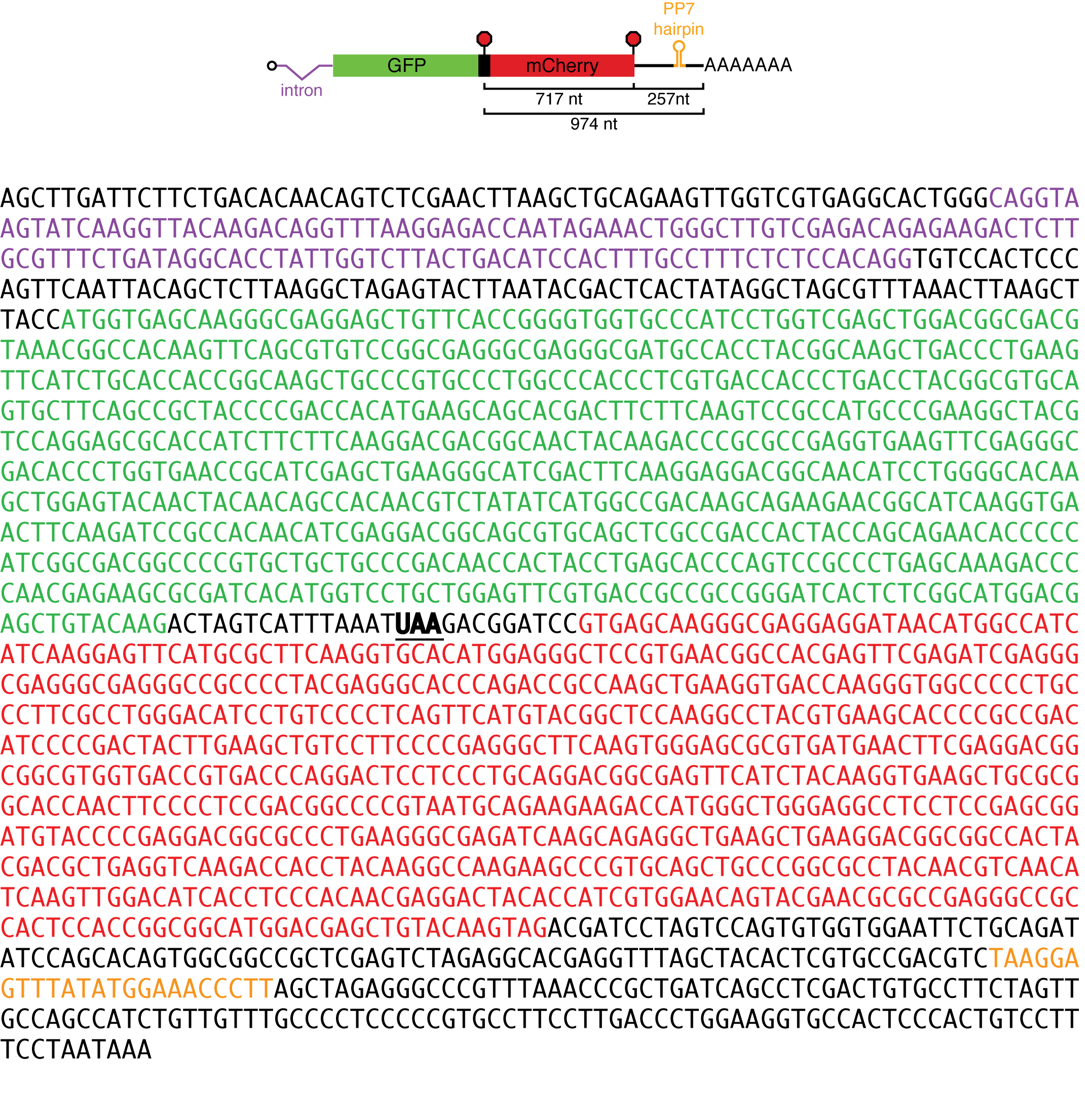

Supplement: S1 Fig — Top, schematic of the dual-fluorescent readthrough and NMD reporters. Bottom, sequence of a reporter gene containing an in-frame UAA termination codon (underlined) between the GFP (green text) and mCherry (red text) ORFs. The intron and PP7 hairpin sequences are indicated in purple and orange, respectively. (TIF) [file pone.0173980.s001.tif]
